# Association of Restless Legs Syndrome With Risk of Suicide and Self-harm

**DOI:** 10.1001/jamanetworkopen.2019.9966

**Published:** 2019-08-23

**Authors:** Sheng Zhuang, Muzi Na, John W. Winkelman, Djibril Ba, Chun-Feng Liu, Guodong Liu, Xiang Gao

**Affiliations:** 1 Department of Neurology, The Second Affiliated Hospital of Soochow University, Suzhou, Jiangsu, China; 2 Department of Nutritional Sciences, Pennsylvania State University, State College, University Park; 3 Department of Psychiatry, Massachusetts General Hospital, Boston; 4 Department of Neurology, Massachusetts General Hospital, Boston; 5 Department of Public Health Sciences, Pennsylvania State University College of Medicine, Hershey

## Abstract

**Importance:**

Restless legs syndrome (RLS) is a common neurologic disorder that has been previously found to be associated with higher odds of suicidal ideation. In the context of the increasing suicide rate in the United States, the evidence regarding the association between RLS and the risk of suicide and self-harm is limited.

**Objective:**

To investigate the association between RLS and risk of suicide and self-harm.

**Design, Setting, and Participants:**

This cohort study was performed using Truven Health MarketScan national claims data from 2006 to 2014; the baseline data were from 2006 to 2008, and the follow-up data covered 6 years (January 1, 2009, to December 31, 2014). Included were 24 179 nonpregnant participants with RLS and 145 194 age- and sex-matched participants without RLS at baseline (2006-2008), who were free of suicide, self-harm, cardiovascular disease, or cancer at study baseline. Data analysis was performed from February 1, 2018, to January 1, 2019.

**Exposure:**

Diagnosis of RLS, as identified by the *International Classification of Diseases*, *Ninth Revision* code.

**Main Outcomes and Measures:**

Incident suicide and self-harm event, identified by the *International Classification of Diseases*, *Ninth Revision* diagnosis code.

**Results:**

Among 169 373 participants in the current analysis, the mean (SD) age was 49.4 (9.1) years; 53 426 (31.5%) participants were men. During a mean (SD) follow-up duration of 5.2 (2.2) years, 119 incident suicide and self-harm cases were identified. Individuals with RLS had a higher risk of suicide or self-harm compared with those without RLS (adjusted hazard ratio, 2.66; 95% CI, 1.70-4.15), after adjusting for lifestyle factors (eg, alcohol and obesity), presence of chronic diseases (eg, depression, insomnia, diabetes, chronic kidney disease, peripheral neuropathy, iron-deficiency anemia, and Parkinson disease), and use of medications. Excluding those with depression, insomnia, obstructive sleep apnea, and other common chronic conditions, the significant association between RLS and suicide or self-harm persisted (adjusted hazard ratio, 4.14; 95% CI, 2.17-7.92).

**Conclusions and Relevance:**

Restless legs syndrome was associated with a high risk of suicide and self-harm, and the risk was independent of most identified diseases and conditions.

## Key Points

**Questions:**

Is restless legs syndrome associated with a high risk of suicide and self-harm?

**Findings:**

In a cohort study that included 169 373 participants, individuals with restless legs syndrome had a higher risk of suicide and self-harm compared with age- and sex-matched participants without restless legs syndrome, and the increased risk was independent of common diseases and conditions.

**Meaning:**

Restless legs syndrome was associated with an increased risk of suicide and self-harm.

## Teaser

This cohort study of commercially insured US individuals examines the association between restless legs syndrome and risk of suicide and self-harm.

## Introduction

Restless legs syndrome (RLS), which affects approximately 5% of the population in Western countries, is a sensorimotor disorder characterized by an unstoppable desire to move the legs and is usually accompanied by irritation in the lower extremities.^1^ Although the mechanisms are not fully understood, RLS is associated with many physical and mental conditions, including poor sleep,^2,3,4^ depression,^5,6,7^ cardiovascular diseases,^8,9,10^ attention-deficit/hyperactivity disorder,^11^ obesity,^12^ and diabetes,^13^ and leads to a significant reduction in the quality of life.^2,3,4,14,15^ In the general population, a 30% to 90% increase in the risk of mortality has been reported in association with RLS.^16,17,18^ The association may occur through pathways other than sleep deprivation and/or sleep disorders, including several common lifestyle risk factors that have been examined elsewhere.^16,17^

Suicide is a global health concern and is associated with multiple risk factors, including male sex, family history of suicide, childhood adversity, alcohol abuse, psychiatric disorders, and sleep problems.^19^ Given that sleep disturbance and depression are highly concurrent in individuals with RLS,^5,6,7,20^ it is plausible that part of the elevated overall mortality risk associated with RLS may be driven by increased risk of death from suicide.

To date, few studies^5,21,22^ have examined the association between RLS and suicidal ideation or attempt. These studies were mainly conducted in small samples of fewer than 200 individuals with RLS and seldom controlled for the confounding conditions of depression, chronic diseases, and medication use. In the context of the high prevalence of RLS and increasing suicide rate in the United States,^23^ addressing the potential association between RLS and suicide is timely. Using longitudinal medical claim data from a large sample of commercially insured patients in the United States, we investigated the association between RLS and risk of suicide and self-harm and hypothesized that individuals with RLS have a higher risk of suicide and self-harm compared with those without RLS.

## Methods

### Data Source

We performed this cohort analysis using data from 2006 to 2014 from the Truven Health MarketScan Commercial Claims and Encounters database. The database includes data for 240 million unique individuals younger than 65 years who were enrolled in commercially insured medical, pharmacy, and dental plans in all 50 states of the United States.^24,25^ Data are contributed by a convenience sample from multiple sources (employers, health plans, and state Medicaid agencies) and represent fully paid and adjudicated claims for inpatient and outpatient services and outpatient prescription drugs. Longitudinal tracking of patient-level health care information provides comprehensive data, including demographic characteristics, diagnosis, treatments, procedures, and costs. A unique identifier is assigned to each person in the database before creating links to individual data of different years or types, and the data are collected for annual releases when all claims have been paid, which adds to the accuracy and completeness of the data source. To ensure privacy and quality of the database, all data are deidentified. Diagnosis and procedure codes are compared with the codes that were in effect when the raw data were collected and are edited if necessary. More information about the database is published elsewhere.^24^

This study protocol was submitted to the Pennsylvania State University institutional review board and was not considered to be human subject research, as defined by the US Department of Health and Human Services.^26^ Because data of the study participants were deidentified, informed consent was waived. This study followed the Strengthening the Reporting of Observational Studies in Epidemiology (STROBE) reporting guideline for cohort studies. Data analysis was performed from February 1, 2018, to January 1, 2019.

### Study Population

We included nonpregnant adults aged 20 years and older who had at least 1 inpatient or outpatient medical claim for diagnosed RLS (using *International Classification of Diseases, Ninth Revision *[*ICD-9*] code 333.94) from January 1, 2006, to December 31, 2008. We excluded individuals with suicide or self-harm (*ICD-9* codes E950-E959 and E980-E989), cardiovascular disease, and/or cancer (see *ICD-9* codes in the eTable in the Supplement) before December 31, 2008. There were 24 179 participants with RLS identified according to the inclusion and exclusion criteria. Each participant with RLS was matched to 6 participants without RLS who were free of cardiovascular disease and cancer during 2006 to 2008, according to sex and year of birth.

### Outcome

The outcome of interest was incident suicide and nonfatal self-harm cases, identified by *ICD-9* codes, occurring from January 1, 2009, to December 31, 2014. Suicide and self-harm events were defined by the presence of *ICD-9* codes E950-E959 or as death or injury with undetermined intent (*ICD-9* codes E980-E989) in the database.

### Covariates

In addition to demographic variables, we also extracted the following individual-level data in the database: alcohol consumption (based on self-report; yes or no), presence of chronic obstructive pulmonary disease (COPD; *ICD-9* codes 490-496), presence of other chronic diseases or conditions (diabetes, hypertension, obesity, hyperlipidemia, obstructive sleep apnea [OSA], depression, insomnia, peripheral neuropathy, rheumatologic disease, osteoarthritis, iron-deficiency anemia, chronic kidney disease, or Parkinson disease; see the *ICD-9* codes in the eTable in the Supplement), and medication history (antidepressant, antiplatelet, anticoagulant, statin, nonstatin, antihypertensive, or antidiabetic drugs). We used diagnosis of COPD as a surrogate for smoking because tobacco consumption was not available in the database. All chronic diseases and conditions were reported by physicians and were assessed only in 2006 to 2008.

### Statistical Analysis

SAS statistical software version 9.4 (SAS Institute) was used to perform all statistical analysis using 2-tailed *P* < .05 as the significance level. For each participant, person-years were calculated from the date of RLS diagnosis to the date of suicide or self-harm, the date lost to follow-up, or December 31, 2014, whichever date came first. The presence of an RLS diagnosis was deemed as the primary exposure.

The Cox proportional hazards model was applied to calculate the hazard ratio (HR) and 95% confidence interval for each group of the cohort, and the proportional hazards assumption was satisfied. In the multivariable models, we adjusted for residence (rural or urban), region (South, West, Midwest, Northeast, or unknown), alcohol consumption (yes or no), obesity, and presence of COPD. We further adjusted for the presence of diabetes, hypertension, hyperlipidemia, sleep apnea, depression, insomnia, peripheral neuropathy, rheumatoid arthritis, osteoarthritis, chronic kidney disease, iron-deficiency anemia, or Parkinson disease (each yes or no), and use of antidepressant, antiplatelet, anticoagulant, statin, antihypertensive, and antidiabetic drugs (each yes or no). In a secondary analysis, we also included 16 suicide or self-harm cases occurring during 2006 to 2008 but after the RLS index date. We also tested potential interactions of the presence of RLS (yes or no) with age and sex (female or male) in the Cox models with adjustment for the aforementioned confounders. To understand the short- vs long-term association of RLS with suicide and self-harm risk, we separately calculated HRs of suicide that occurred in the first and in the last 3 years (midpoint of RLS follow-up duration) of RLS diagnosis, adjusting for the preceding covariates. To understand whether medication use associated with RLS modifies the potential association between RLS and suicide or self-harm, we further categorized participants with RLS into 2 groups: 16 684 individuals with RLS receiving treatment (including dopaminergic agents, benzodiazepines, anticonvulsants, and opiates) and 7495 individuals with RLS not receiving treatment. We reexamined and compared suicide and self-harm risk in individuals with no RLS (reference group), individuals with RLS receiving treatment, and individuals with RLS not receiving treatment.

Considering the comorbid diseases and conditions frequently associated with RLS, we performed several sensitivity analyses to minimize potential confounding bias. First, we performed analysis with inverse probability of RLS weighting using the propensity score, which was calculated using the aforementioned covariates in the final model, to balance baseline data between individuals with and without RLS. Second, because depression, use of antidepressant medications, and common sleep disorders, such as insomnia and OSA, are strongly associated with both RLS and suicide,^5,6,20,27,28^ we repeated analyses by excluding individuals with depression or antidepressant medication use and individuals with insomnia or OSA, respectively. Finally, we reran the analyses for an apparently healthy population after excluding 60 039 participants with obesity, COPD, diabetes, hypertension, hyperlipidemia, OSA, depression, insomnia, peripheral neuropathy, rheumatologic disease, osteoarthritis, iron-deficiency anemia, chronic kidney disease, and Parkinson disease.

## Results

In total, 169 373 individuals (mean [SD] age, 49.4 [9.1] years; 53 426 [31.5%] male) accumulated a total of 874 314 person-years through the study period. The 24 179 participants with RLS were individually matched on a 1:6 ratio with 145 194 participants without RLS who were included in the final analysis. Compared with individuals without RLS, higher proportions of individuals with RLS lived in a rural area, had COPD, had other chronic disease or conditions (eg, diabetes, hypertension, hyperlipidemia, OSA, insomnia, and osteoarthritis), and consumed alcohol in 2006 to 2008 (Table 1). Depression and concomitant use of antidepressant medications were more common among participants with RLS versus those without RLS, suggesting relatively poorer mental health status.

**Table 1.** Baseline Characteristics of Participants by RLS Status

Characteristic	Participants, No. (%)		*P* Value
	No RLS (n = 145 194)	RLS (n = 24 179)	
Age, mean (SD), y	49.4 (9.1)	49.4 (9.1)	
Male	45 798 (31.5)	7628 (31.5)	
Rural residence	28 115 (19.4)	5994 (24.8)	<.001
Region of United States			
South	58 133 (40.0)	12 330 (51.0)	<.001
West	32 758 (22.6)	3104 (12.8)	
Midwest	40 088 (27.6)	7245 (30.0)	
Northeast	13 997 (9.6)	1448 (6.0)	
Unknown	218 (0.2)	52 (0.2)	
Alcohol consumption	422 (0.3)	150 (0.6)	<.001
Baseline comorbidities			
Diabetes	10 625 (7.3)	2365 (9.8)	<.001
Hypertension	27 849 (19.1)	6619 (27.4)	<.001
Obesity	2148 (1.5)	1151 (4.8)	<.001
Hyperlipidemia	3973 (2.7)	1113 (4.6)	<.001
Obstructive sleep apnea	2507 (1.7)	3191 (13.2)	<.001
Depression	7648 (5.3)	3949 (16.3)	<.001
Insomnia	1537 (1.1)	1651 (6.8)	<.001
Peripheral neuropathy	566 (0.4)	626 (2.6)	<.001
Rheumatologic disease	1557 (1.1)	486 (2.0)	<.001
Osteoarthritis	7079 (4.9)	2998 (12.4)	<.001
Iron-deficiency anemia	1480 (1.0)	659 (2.7)	<.001
Chronic kidney disease	372 (0.3)	122 (0.5)	<.001
Parkinson disease	84 (0.1)	55 (0.2)	<.001
Chronic obstructive pulmonary disease	8769 (6.0)	3301 (13.7)	<.001
Baseline medications			
Antidepressant	25 663 (17.7)	7326 (30.3)	<.001
Antiplatelet	284 (0.2)	76 (0.3)	<.001
Anticoagulant	28 (0.02)	2 (0.01)	.30
Statin	20 197 (13.9)	2759 (11.4)	<.001
Nonstatin	6745 (4.6)	1127 (4.7)	.91
Antihypertensive	40 380 (27.8)	5899 (24.4)	<.001
Antidiabetic	15 855 (10.9)	2197 (9.1)	<.001

Abbreviation: RLS, restless legs syndrome.

During the 6-year follow-up period (January 1, 2009, to December 31, 2014; mean [SD], 5.2 [2.2] years), we identified 119 incident cases of suicide and self-harm. In the age- and sex-adjusted model (model 1), individuals with RLS had a higher risk of suicide and self-harm compared with those without RLS (adjusted HR, 3.55; 95% CI, 2.38-5.30; Table 2). Further adjustment for other potential confounders, including residence, region, alcohol consumption, obesity, presence of major chronic diseases, and medication use, the association between RLS and suicide and self-harm risk attenuated but remained significant (adjusted HR, 2.66; 95% CI, 1.70-4.15; Table 2). Further inclusion of 16 incident cases of suicide or self-harm during 2006 to 2008, occurring after the RLS index date, generated similar significant results (adjusted HR, 3.98; 95% CI, 2.68-5.91). We observed a similar 2 to 3 times higher suicide and self-harm risk in participants who received a diagnosis of RLS during the first 3 years (adjusted HR, 3.11; 95% CI, 1.73-5.60) or during the last 3 years of the follow-up period (adjusted HR, 2.10; 95% CI, 1.05-4.20). Compared with participants without RLS, a significantly higher risk of suicide and self-harm was observed both among participants with RLS receiving treatment (adjusted HR, 2.57; 95% CI, 1.60-4.15) and those with RLS not receiving prescribed treatment (adjusted HR, 3.10; 95% CI, 1.29-7.40). A 3- to 4-fold increase in the suicide and self-harm risk was consistently observed in the sensitivity analyses using propensity score matching on the baseline data (adjusted HR, 4.28; 95% CI, 3.15-5.80), in participants without depression or use of antidepressant medication (adjusted HR, 3.75; 95% CI, 2.08-6.77), in participants without insomnia or OSA (adjusted HR, 3.18; 95% CI, 1.99-5.08), and in apparently healthy participants (adjusted HR, 4.14; 95% CI, 2.17-7.92; Table 3). We did not find a significant interaction association between RLS and age or between RLS and sex on suicide and self-harm risk.

**Table 2.** Adjusted Models for Suicide and Self-harm, by RLS Status

Variable	No RLS	RLS
Cases, No.	85	34
Person-years, No.	775 879	98 435
Incident rate (cases per 1000 person-years)	0.11	0.35
Model, hazard ratio (95% CI)		
Model 1^a^	1 [Reference]	3.55 (2.38-5.30)
Model 2^b^	1 [Reference]	2.66 (1.70-4.15)

Abbreviation: RLS, restless legs syndrome.

^a^ Model 1 was adjusted for age and sex.

^b^ Model 2 was adjusted for age, sex, residence (rural or urban), region (South, West, Midwest, Northeast, or unknown), alcohol consumption, and obesity; the presence of chronic obstructive pulmonary disease (surrogate for smoking), diabetes, hypertension, hyperlipidemia, obstructive sleep apnea, depression, insomnia, peripheral neuropathy, rheumatic arthritis, osteoarthritis, chronic kidney disease, iron-deficiency anemia, or Parkinson disease (each, yes or no); and use of antidepressant, antiplatelet, anticoagulant, statin, antihypertensive, and antidiabetic drugs (each, yes or no).

**Table 3.** Sensitivity Analysis for Suicide and Self-harm, by RLS Status

Variable	HR (95% CI)	
	No RLS	RLS
Propensity score adjustment^a^	1 [Reference]	4.28 (3.15-5.80)
Excluding participants with		
Depression or antidepressants use	1 [Reference]	3.75 (2.08-6.77)
Insomnia or obstructive sleep apnea	1 [Reference]	3.18 (1.99-5.08)
Common chronic disease conditions^b^	1 [Reference]	4.14 (2.17-7.92)

Abbreviations: HR, hazard ratio; RLS, restless legs syndrome.

^a^ Adjusted for age, sex, residence (rural or urban), region (South, West, Midwest, Northeast, or unknown), alcohol consumption, and obesity; presence of chronic obstructive pulmonary disease (surrogate for smoking), diabetes, hypertension, hyperlipidemia, sleep apnea, depression, insomnia, peripheral neuropathy, rheumatic arthritis, osteoarthritis, chronic kidney disease, iron-deficiency anemia, or Parkinson disease (each, yes or no); and use of antidepressant, antiplatelet, anticoagulant, statin, antihypertensive, and antidiabetic drugs (each, yes or no).

^b^ Common chronic disease conditions included obesity, chronic obstructive pulmonary disease, diabetes, hypertension, hyperlipidemia, sleep apnea, depression, insomnia, peripheral neuropathy, rheumatic arthritis, osteoarthritis, chronic kidney disease, iron-deficiency anemia, and Parkinson disease.

## Discussion

In this cohort of 169 373 participants in the United States, we found that individuals with RLS had a higher risk of suicide and self-harm than did those without RLS. The association was independent of age, sex, geographic differences, depression, sleep disorders, other chronic medical conditions, and medication use.

The absolute event rate, with 119 documented cases across 169 373 participants, seemed low but may align reasonably with the reported increasing suicide rates in the United States.^23^ However, current end points were a mix of suicide and self-harm events; of note, a higher lifetime nonfatal self-harm rate was reported in approximately 4% to 6% of US adults, with most not receiving medical help.^29,30^ Therefore, one should keep in mind that the self-harm cases in particular could not be fully identified in the claims database representing insured patients. Because of the etiologic heterogeneity of suicide and self-harm, a thorough history-taking and mental health screening are important in the clinical setting to capture underlying suicide risk factors for individuals with RLS. Early psychological intervention may be necessary, and relevant medications should be tailored and monitored on usage and dosage^31^ for potential adverse effects that may induce suicidal ideation.^32,33,34^

Associations between RLS and suicide have been rarely examined previously. In a case-control study including 130 individuals with RLS and 2265 controls in a German community, Winkelmann et al^5^ found that 34.8% of individuals with RLS reported suicidal thoughts, compared with 2.6% of controls. A 2016 study^22^ of 126 psychiatrically hospitalized Lebanese patients found that one-fifth of patients with suicidal thoughts had RLS symptoms. However, self-reported suicidal ideation was identified with a single question in this study, and ideation is not synonymous with true suicide or self-harm attempt. One recent case-control study^21^ of RLS further targeted suicidal ideation and behavior with more comprehensive assessment of 4 questions among 192 individuals with moderate-to-severe RLS. The study^21^ reported a significantly higher prevalence of suicidality for the RLS group compared with the 158 controls without RLS (27.1% vs 7.0%) and found that individuals with RLS had a 3 times higher odds of lifetime suicidal ideation or behavior. Consistent with our study, they also found that the association between RLS and suicide was independent of depression and other covariates (age, sex, race, marital status, education, income, and drug and alcohol abuse history). In sum, the few existing studies looking at the association between RLS and suicide were retrospective in design, conducted in small samples, and focused on suicidal thoughts or ideation, rather than suicide and self-harm events.

The underlying mechanisms of the association between RLS and suicide or self-harm are still unclear. A commonly accepted explanation is the potential mediating role of depression, which is associated with both RLS and suicide. Compared with participants without RLS, symptoms of depression were reported or diagnosed more frequently among participants with RLS.^5,6,7,35,36^ Depressive symptoms and RLS are also reported to be positively associated in 1 cross-sectional study^35^ of primary care patients. In our study, however, we found that the elevated risk of suicide and self-harm in those with RLS was independent of depression—that is, the significant association remained when we adjusted for depression and among participants with no depression. In our study, depression was diagnosed and confirmed by the *ICD*-9 code; thus, we did not include undiagnosed depression cases. We cannot rule out the possibilities that the seemingly direct association between RLS and self-harm was still partially dependent on depression symptoms that were mild or undetected. In addition, even when they are free of comorbid psychiatric disorders, individuals with RLS exhibit high neuroticism in several dimensions.^37,38,39^ This personality trait has a robust association with suicide^40^ by prospectively contributing to the development of depressive symptoms.^41^

Comorbid sleep problems (eg, sleep disturbance, insomnia, and OSA) may also contribute to elevated suicide risk in individuals with RLS. After summarizing data from 39 studies, a recent meta-analysis^42^ reported that both general sleep disturbance and insomnia were associated with a 2-fold increase in the risk of completed suicide. After controlling for key covariates, OSA was reported to be associated with 1.5 times higher odds of suicidal ideation and suicide planning in a secondary analysis of nationally representative US adults.^43^ Obstructive sleep apnea was reported to co-occur in 32% to 57.5% of individuals with RLS in previous studies,^44,45,46^ and treatment for OSA even benefited RLS symptoms in 20 of 26 patients.^46^ Nevertheless, research focusing on these sleep disorders and suicide in individuals with RLS is still scarce. Shared neurobiological^47,48^ and genetic^49^ bases for RLS and sleep disorders have been suggested, which may partially explain the common comorbidity. Brainstem sleep regulating centers^47^ and dopaminergic pathway^48^ could be dysfunctional for both patients with RLS and OSA. In a recent genome-wide association study, the *MEIS1* polymorphism highlighted the genetic link between insomnia and RLS.^49^ In our study, we did not observe a significant association of insomnia or OSA and RLS with suicide and self-harm risk, indicating that sleep problems may not fully explicate the association between RLS and suicide.

Strengths of our study include an analysis based on longitudinal data of a large sample of individuals with RLS and controls without RLS with a long follow-up time (mean [SD], 5.2 [2.2] years). The prospective design of the current study provided unique opportunities to examine the temporal association between RLS and suicide that previous studies with cross-sectional or case-control designs could not achieve. Furthermore, we used suicide and self-harm rather than suicide ideation as our end point of interest, which avoids bias in the retrospective reporting of suicidal ideation.

### Limitations

Several limitations need to be noted when interpreting our findings. First, we may have misclassified suicide or self-harm and other medical conditions by using *ICD-9* codes instead of newer *ICD* codes. For suicide or self-harm coding, the *ICD-9* and *International Classification of Diseases*,* Tenth Revision* codes may yield different national statistics^50^ and trends data,^51,52^ although that coding transition did not alter the estimates of suicide-associated causes of death.^43,44,45,46^ As mentioned before, because the *ICD-9* codes were derived from claim documents, we may have underestimated the prevalence of suicide or self-harm without the inclusion of patients who did not seek care. Misclassification may occur if errors in *ICD-9* coding were not randomly distributed among physicians, clinics, and hospitals. Because identical codes were used, the 119 cases identified in the data set were a combination of completed suicide and nonfatal self-harm cases, which prevented us from distinguishing the 2 groups of people for further analysis. Second, although the association between RLS and suicide persisted in the series of models with multiple adjustments and in the set of sensitivity analyses, several available covariates were still limited because of the absence of a direct measure of smoking (we used the presence of COPD as a surrogate measure of smoking), amount of alcohol consumption, and severity of depression. Residual confounding could exist because we did not have data on some important factors, such as educational level, family history, and recent stressful life events. The frequency of major chronic diseases (eg, hypertension and other sleep disorders) could also be underreported, which is another potential source for residual confounding. More studies with comprehensive assessment of existing and underlying covariates would be meaningful to confirm our findings. Third, the data set consists of a convenience sample of participants who are younger than 65 years, commercially insured, and primarily work for large employers in the United States. The generalizability of our study findings to other populations is unclear.

## Conclusions

This study suggests that individuals with RLS have a higher risk of committing suicide and self-harm, and the increased risk is independent of depression, sleep disorders, chronic conditions, and other factors being examined. Future investigations are needed to explore the possible mechanisms by which RLS increases suicide risk. Given the high prevalence of RLS and increasing incidence of suicide in the United States, it may be critical to consider assessment for suicide risk factors and suicidal ideation, as well as potential psychological interventions and suicidal management in the treatment of individuals with RLS.

## Notes

Supplement. **eTable.** *ICD-9* Codes for Diseases and Conditions
