# Supplementary material for: Association of Restless Legs Syndrome With Risk of Suicide and Self-harm
Source: JAMA Netw Open. 2019 Aug 23;2(8):e199966. doi: 10.1001/jamanetworkopen.2019.9966 (PMC6714009; doi:10.1001/jamanetworkopen.2019.9966)
Supplement: Supplement. — eTable. ICD-9 Codes for Diseases and Conditions [file jamanetwopen-2-e199966-s001.pdf]

Supplementary Online Content

Zhuang S, Na M, Winkelman JW, et al. Association of restless legs syndrome with risk of suicide and self-harm. *JAMA Netw Open*. 2019;2(8):e199966. doi:10.1001/jamanetworkopen.2019.9966

eTable. *ICD-9* Codes for Diseases and Conditions

This supplementary material has been provided by the authors to give readers additional information about their work.

© 2019 Zhuang S et al. *JAMA Network Open.*

eTable. *ICD-9* Codes for Diseases and Conditions

Name	ICD-9 code
CVD	36.0[a], 36.1[a], 410-413, 414.0, 414.8, 414.9, 428, 430-438
Cancer	140-172, 174-199.1, 200-208
Diabetes	250
Hypertension	401-405
Obesity	278.0
Hyperlipidemia	272.2
OSA	327.2x, 780.57
Depression	296.2, 296.3, 296.5, 296.6, 296.89, 300.4, 309.0, 309.1, 311
Insomnia	307.42, 780.52
Peripheral neuropathy	356.x
Rheumatologic disease	710.0, 710.1, 710.4, 714.0-714.2, 714.81, 725
Osteoarthritis	715.x
Iron deficiency anemia	280
CKD	585.x
Parkinson's disease	332

Abbreviations: ICD-9: International Classification of Diseases, Ninth Revision; CVD: cardiovascular disease; OSA: obstructive sleep apnea; CKD: chronic kidney disease.

[a] Identified by ICD-9 Clinical Modification Procedure Codes.

© 2019 Zhuang S et al. *JAMA Network Open.*
